# Is “Self-Experience” Really a Transdiagnostic Concept? Preliminary Evidence in Favor of the Transdiagnostic Conception of Functional Analytic Psychotherapy

**DOI:** 10.3389/fpsyt.2021.671223

**Published:** 2021-08-24

**Authors:** Ana María González Palma, Laura C. Sánchez-Sánchez, José Manuel Garcia-Montes

**Affiliations:** ^1^Department of Psychology, University of Almería, Almería, Spain; ^2^Department of Developmental and Educational Psychology, University of Granada, Granada, Spain

**Keywords:** self, psychopathology, transdiagnostic, functional analytic psychotherapy, development

## Abstract

Functional Analytic Psychotherapy (FAP) offers a radically behavioral and transdiagnostic conception of the formation of the “self” and the appearance of a diversity of psychological problems. This study examined the extent to which a wide variety of psychological disorders (somatization, obsessive–compulsive, interpersonal sensitivity, depression, anxiety, hostility/aggressiveness, phobic anxiety, paranoid ideation, and psychoticism) and a global index of psychopathological severity may in fact be linked to problems of the “self” according to the FAP conception. Two questionnaires, one related to self-experience according to FAP and the other to find the scores on several different psychopathology scales, were administered to 280 adult Spaniards for this purpose. The results confirmed the transdiagnostic nature of the “self”-experience. There are significant and strong correlations between all the psychopathology scales studied and self-experience. Linear regression analyses also show that, along with age and gender, in some cases, the score on self-experience predicts each and every one of the psychopathological variables studied, in addition to the Global Severity Index. These results are discussed and related to the transdiagnostic approach to psychopathology.

## Introduction

Kohlenberg and Tsai (1, 2), based on various studies by Skinner (3–6), developed a radically behavioral conception of development of the “self.” According to their proposal, the verbal report “I” emerges as an independent functional unit starting from learning larger verbal expressions in which the expression “I” is used (2). Therefore, in technical terms of behavior analysis, the subjective experience of the “self” would be a question of “self-tact” (7). Learning the “self”-experience as self-tact would be similar to learning tacts referring to the properties of stimuli (e.g., colors), in which based on multiple examples with different objects where all the stimuli are different except the color, the speaker learns to develop the functional unit “red” or “blue” from wider verbal expressions learned beforehand (“red car,” “red apple,” “blue window,” “blue water,” etc.). The main difference between learning colors as small functional units and learning the “self” as a small functional unit is that the first are completely public stimuli, while the second is a private experience (8). According to Skinner (5), the verbal community teaches children to tact private events indirectly by using public accompaniments of the private events, which can generate certain problems due, for example, to the difficulty in accessing the experience they intend to tact or else the interest of the parents themselves in labeling certain types of experiences in their children (and not others). Kohlenberg and Tsai (1) proposed that the “self” experience emerges as a functional unit from the acquisition of larger units as a child learns to speak and showed that this process occurs in three stages. In Stage I, which usually takes place during the first 2 years of life, the child learns large functional units, such as, “I have ice cream” or “I want candy.” These functional units are learned as a whole. During Stage II, smaller functional units appear when the common element in the large functional units from the previous stage is selected (e.g., “I have” or “I want”), which may be combined with different objects. Finally, during Stage III, as the only element present in all the functional units in Stage II, the functional unit “I” would appear at the same time as one experiences oneself. Although the process is similar to the one in Stage II, it should be noted that the experience of “I” in Stage III should be completely under the control of private stimuli. Therefore, during successive stages, one goes from public to private control of the “self” experience, which should take its complete form in Stage III.

Tsai et al. (8) suggested that self-experiences may be conceptualized as varying on a continuum from complete private control over the experience (in which case a strong self would be generated) to complete public control (which would generate psychological problems, the stronger public control, the more severe the problems). As Ferro-García and Valero-Aguayo (9) stated so well, the formation of the self and its possible problems would then be a transdiagnostic hypothesis as proposed by Functional Analytic Psychotherapy (FAP).

In a similar line, the authors of *Acceptance and Commitment Therapy* [ACT; (10)], a similar approach originating from radical Skinnerian behaviorism, have proposed that the construction of a sense of self, which allows the person to assume any aspect of the experience as his or her own even though this be negative or adverse and, in time, not become trapped by such experiences would constitute an important protective factor of psychological health for individuals (11).

Indeed, Kohlenberg and Tsai already proposed such a transdiagnostic conception of psychological problems in 1991, even before the term was used in the influential article by Fariburn et al. (12), in which they proposed a series of processes common to different eating disorders.

In spite of its interest, and the progress it led to at the time, as far as we know, the FAP approach to understanding the development of mental disorders has hardly been subjected to testing, with the notable exception of the work of Kanter et al. (13). Growing attention and controversy that transdiagnostic approaches are now receiving in mental health (14, 15) have renewed interest in the proposal by Kohlenberg and Tsai (1). The term “transdiagnostic” has been used to appoint the psychological processes involved in different disorders, as therapeutic approximations that address psychopathology across diagnostic boundaries, allowing them to target comorbid conditions and reduce therapist training burden (16). The proposal by Kohlenberg and Tsai (1) about the problems that may arise in the development of “self” describes functionally causal mechanisms that inform the development of classes of disorders and can be targeted in treatment (16, 17), supposing in this way a fully transdiagnostic vision with a foundation in the development of private experience. Thus, Basten and Touyz (18) recently emphasized that although the sense of self is a cornerstone of psychological inquiry and therapy and a defining feature of a range of psychopathological conditions, it is poorly understood. This lack of understanding may be due to the many diverse theoretical perspectives that attempt to explain the development of the sense of self and that, in turn, it has been divided into a series of different aspects (agency, continuity, coherence, completeness, authenticity, vitality, etc.) that would each require separate research (18).

The advantage of Kohlenberg and Tsai's proposal on the sense of self as a transdiagnostic factor stems precisely from its parsimonious integrating nature, as FAP integrates both behavioral and psychodynamic facets (19).

The previous investigation has also brought to light the degree of public control over the experience of the self-covaried with self-esteem and dissociation. In addition, a clinical sample diagnosed with borderline personality disorder showed greater public control over the experience of self than did an undergraduate sample (13).

The objective of this study was to find out to what extent problems of the “self” would be involved in different psychological disorders. To do this, we started out from the idea that there would be a strong relationship between “self” disorders and a wide variety of psychological problems.

## Methods

### Participants

The study sample consisted of 280 Spaniards aged 18–68 (*M* = 36.87, *SD* = 11.49), of whom 67.1% were women (*n* = 188) and 32.9% were men (*n* = 92). Here, 90.7% of the sample did not receive any type of psychological treatment, whereas 9.3% underwent psychological therapy. In addition, 5.7% of the sample was prescribed some type of pharmacological treatment for some mental health issues. The sample was selected by non-probabilistic convenience sampling. All participants agreed voluntarily to answer the questionnaires. Among the inclusion criteria was that they be of legal age.

### Variables and Instruments

All the participants answered an online questionnaire containing the following instruments:

#### Experience of Self Scale

This instrument (13) measures the extent of private and public control of the “self”-experience. It is made up of 37 items with a Likert-type response format from 1 (Never) to 7 (Always). It is divided into four subscales that study five “self” experiences (feeling, wants, attitudes, opinions, and actions) according to the nature of the control of experiences and closeness to the other persons. Subscale 1 evaluates the “self”-experiences about oneself in general; Subscale 2 does so with respect to acquaintances (coworkers, neighbors, etc.); in Subscale 3, with regard to a personal relationship (friend, family member, etc.); and Subscale 4 measures “self”-experiences about oneself in relation to other people. This study used the Spanish version by Valero-Aguayo et al. (20). The Cronbach's α for the Spanish version is 0.91. Subscale 4 has the lowest coefficient (α = 0.61), followed by subscale 1 (α = 0.66). The rest of the subscales have a Cronbach's α over 0.90. When reliability is measured using the split-half method, no coefficient is below 0.83.

#### Brief Symptom Inventory

This questionnaire, (21) composed of 53 items, is the short form of the Symptom Checklist-90-Revised [SCL-90-R; (22)] and provides a measure of a person's psychopathological state through symptoms experienced during the last 7 days. The Likert-type answer choices go from 1 (“Not at all”) to 4 (“A lot”). It gives scores on nine dimensions (somatization, obsessive–compulsive, interpersonal sensitivity, depression, anxiety, hostility/aggressiveness, phobic anxiety, paranoid ideation, and psychoticism) and three overall indices [Global Severity Index (GSI), Positive Symptom Total (PST), and Positive Symptom Distress Index (PSDI)], of which the GSI is the best indicator of psychological distress. The BSI shows an acceptable internal consistency ranging from 0.71 to 0.85. Several studies have also found good internal consistency for all nine of the original dimensions with very diverse populations (23).

This study used the Spanish translation by Derogatis (24). The correlations between the different subscales of the questionnaire are usually “moderate-to-high” (23).

#### Sociodemographic Variables

The age and gender of the participants were recorded as demographic variables of interest.

### Procedure

The questionnaire was administered remotely by email, social networks, or messaging services such as WhatsApp. Sampling was by convenience. Participation in the study was voluntary, and participants were ensured confidentiality and anonymity. The questionnaire took ~10 min to answer.

The present study is part of a project of a broader scope that received the approval of the Committee of Bioethics of the University de Almería (UALBIO2018/026).

## Results

Table 1 shows the means and standard deviations of participant ages and test scores.

**Table 1:** Means and standard deviations of age and participant scores on the scales.

	**Age**	**EOSS**	**BSI**									
			**GSI**	**S**	**OC**	**IS**	**D**	**A**	**A/H**	**PA**	**PI**	**P**
Mean	36.86	88.86	1.52	1.38	1.75	1,60	1,66	1,60	1,47	1.33	1.48	1.36
SD	11.51	22.70	0.44	0.48	0.61	0.63	0.62	0.55	0.51	0.46	0.54	0.63

*EOSS, Experience of Self Scale; BSI, Brief Symptom Inventory; GSI, Global Severity Index; S, somatization; OC, obsessive–compulsive; IS, interpersonal sensitivity; D, depression; A, anxiety; A/H, aggressiveness/hostility; PA, phobic anxiety; PI, paranoid ideation; P, psychoticism*.

A correlation analysis was performed to calculate the magnitude of the relationships between self-experience and the BSI psychopathology scales. Table 2 shows the correlations found between the EOSS and the psychopathology scales in the BSI.

**Table 2:** Pearson correlations between the EOSS and the BSI subscales.

	**GSI**	**S**	**OC**	**IS**	**D**	**A**	**A/H**	**PA**	**PI**	**P**
EOSS	0.620^**^	0.300^**^	0.615^**^	0.596^**^	0.611^**^	0.452^**^	0.460^**^	0.437^**^	0.493^**^	0.622^**^

*EOSS, Experience of Self Scale; BSI, Brief Symptom Inventory; GSI, Global Severity Index; S, somatization; OC, obsessive–compulsive; IS, interpersonal sensitivity; D, depression; A, anxiety; A/H, aggressiveness/hostility; PA, phobic anxiety; PI, paranoid ideation; P, psychoticism*.

** *p < 0.000*.

To find out whether, along with the sociodemographic variables considered, the participant EOSS score was able to predict the scores on the various BSI subscales, a series of 10 regression analyses were carried out, one for the GSI and nine for each of the BSI psychopathology subscales. The dependent variable for each of the analyses was the participant score on the corresponding BSI subscale. In all of them, the independent variables were age, gender, and participant EOSS scores. Stepwise regression was used with a probability of *F* < 0.05 for inclusion and of *F* > 0.100 for exclusion.

Table 3 presents the main statistics corresponding to the final models of these 10 linear regression analyses.

**Table 3:** Linear regression analysis for the BSI scales.

**BSI subscales**	**Variables entered**	**Adjusted *R*^**2**^**	**Std. error of estimates**	**β**	***t***	**Sig**.
GSI	EOSS	0.410	0.342	0.592	12.710	0.000
	Age			−0.176	−3.777	0.000
S	EOSS	0.086	0.458	0.300	5.235	0.000
OC	EOSS	0.388	0.484	0.595	12.537	0.000
	Age			−0.124	−2.608	0.010
IS	EOSS	0.384	0.496	0.566	11.888	0.000
	Age			−0.185	−3.882	0.000
D	EOSS	0.436	0.471	0.570	12.500	0.000
	Age			−0.261	−5.718	0.000
A	EOSS	0.220	0.489	0.428	7.998	0.000
	Age			−0.148	−2.768	0.006
A/H	EOSS	0.230	0.449	0.434	8.157	0.000
	Age			−0.159	−2.995	0.003
PA	EOSS	0.198	0.417	0.439	8.179	0.000
	Sex			−0.115	−2.147	0.033
PI	EOSS	0.272	0.459	0.464	8.969	0.000
	Age			−0.165	−3.180	0.002
	Sex			0.115	2.246	0.026
P	EOSS	0.208	0.449	0.593	12.789	0.000
	Age			−0.181	−3.910	0.000

*EOSS, Experience of Self Scale; BSI, Brief Symptom Inventory; GSI, Global Severity Index; S, somatization; OC, obsessive–compulsive; IS, interpersonal sensitivity; D, depression; A, anxiety; A/H, aggressiveness/hostility; PA, phobic anxiety; PI, paranoid ideation; P, psychoticism*.

It should be mentioned that the final 10 regression equations were statistically significant. In the first place, for the GSI, *F*_(2,277)_ = 98.13, *p* < 0.01. Similarly, for the somatization subscale, *F*_(1, 278)_ = 27.405, *p* = 0.000; for obsessive–compulsive, *F*_(2,277)_ = 168.745, *p* = 0.000; for interpersonal sensitivity, *F*_(2,277)_ = 87.860, *p* = 0.000; for depression, *F*_(2,277)_ = 108.739, *p* = 0.000; for the anxiety subscale, *F*_(2,277)_ = 40.405, *p* = 0.000; for aggressiveness–hostility, *F*_(2,277)_ = 42.774, *p* = 0.000; for the phobic anxiety subscale, *F*_(2,277)_ = 35.479, *p* = 0.000; for paranoid ideation, *F*_(3,276)_ = 35.708, *p* = 0.000; and finally, for psychoticism, *F*_(2,277)_ = 100.022, *p* = 0.000. In all the equations, the participant's score on the EOSS was entered first. The validity of each of the models was tested using the Durbin–Watson test for independence of errors. The values of all of the models were between 1.5 and 2.5, and thus, independence of residuals may be assumed.

## Discussion

This study addressed the possible transdiagnostic nature of self-experience according to the FAP model. The data found backed the starting hypothesis, and therefore, we think self-experience may be a variable related to very different psychological problems, at least, exactly how they are measured by the BSI. It is worth mentioning that the percentage of explained variance of each of the subscales was considerably high, with the exception of the one on somatization. With regard to this result, the somatization scale reflects perceptions of bodily dysfunction. Examples of items in this factor could be “hot or cold spells,” “nausea or stomach disorders,” and “faintness or dizziness” ((25), p. 244). It is therefore possible that participants who mentioned these symptoms were suffering from some type of somatic disorder that could explain that such experiences occur without having to refer to somatization, and therefore, the minor role of self-experience as a psychological factor involved.

We also think that the data found can back, at least to a certain extent, that the stronger problems with self-experience, the more severe the psychopathology is, as reflected in the strong relationships between the EOSS and the GSI. Likewise, the correlations between the EOSS and the most severe psychopathology scales, such as psychoticism or obsessive–compulsive symptoms, are higher than those with other less severe scales, such as somatization or anxiety, which also backs the dimensional character of the “self-experience” as a transdiagnostic factor. This result is consistent with the results found by Kanter et al. (13) by finding that a clinical sample of clients with a diagnosis of borderline personality disorder showed greater public control over the experience of self than did an undergraduate sample. Similarly, although Kanter et al. (13) did not directly investigate whether the experience of self might be a transdiagnostic factor, they did find a sample in the non-clinical population in the same way as shown in the participants in this investigation that the score in the EOSS correlated positively with dissociation and negatively with self-esteem that, at the same time, can be considered as factors involved in diverse psychological disorders.

Concerning the transdiagnostic approach in the scope of mental health, Fusar-Poli et al. (15) mentioned that most of the research has remained almost entirely confined within the restricted original area of interest of anxiety and depressive disorders.

This preliminary study attempted to go further and find out to what point different diagnoses could be related to self-experience problems. This does not mean that certain factors peculiar to each diagnosis that may influence their development or maintenance cannot interact with transdiagnostic variables common to various disorders (26).

When interpreting these results, it should be taken into account that the analyses used are merely correlational, and therefore, it is not possible to establish causal relationships between the variables studied. To be able to determine the construction of a weak self that leads to scoring high on certain psychopathological symptoms, longitudinal studies would have to be undertaken. It should be noted that this work is merely cross-sectional, with only one measurement point in time, whereas the concept of self-experience is inherently based on a developmental perspective, requiring a longitudinal approach. It should also be considered that the sample was a non-clinical population, and therefore, generalization to the clinical population should be done with all possible precautions. This said, it should also be mentioned that the transdiagnostic conception moves, in and of itself, on a continuum (14), and therefore, the data found in a non-clinical population could be of interest.

It should also be considered that the results found by some researchers on the factor structure of the BSI place the supposed multidimensionality of the questionnaire in doubt (27, 28). Intercorrelations between BSI subscales are usually high. Owing to this, the emphasis on nine distinct subscales of the BSI could be overstated, so recent studies have proposed a two-factor BSI structure; however, the nine-factor model has also had an acceptable level of fit (29). In any case, it should be taken into consideration that the BSI has been shown to be an instrument with good sensitivity, specificity, and positive and negative predictive values (30) and that the nine-factor structure has also been confirmed in a Spanish population (23).

Future lines of work could be directed at a deeper study, through semistructured interviews, in the self-experience characterizing each type of disorder, similar to what has been done in the field of psychosis (31). It would also be of interest to implement interventions directed specifically at strengthening the sense of “self” in patients, combining the FAP itself with techniques from other third-wave approaches, such as validation (32, 33). In any case, it opens the possibility of studying the development of the self according to practices in the construction of the private experience and its influence in the development of psychological disorders, enabling a line of work focusing on prevention (34).

## Data Availability Statement

The raw data supporting the conclusions of this article will be made available by the authors, without undue reservation.

## Ethics Statement

The studies involving human participants were reviewed and approved by UALBIO2018/026. The patients/participants provided their written informed consent to participate in this study.

## Author Contributions

AG and JG contributed to conception and design of the study. JG performed the statistical analysis. AG wrote the first draft of the manuscript. All authors contributed to manuscript writting, revision, read, and approved the submitted version.

## Conflict of Interest

The authors declare that the research was conducted in the absence of any commercial or financial relationships that could be construed as a potential conflict of interest.

## Publisher's Note

All claims expressed in this article are solely those of the authors and do not necessarily represent those of their affiliated organizations, or those of the publisher, the editors and the reviewers. Any product that may be evaluated in this article, or claim that may be made by its manufacturer, is not guaranteed or endorsed by the publisher.
